# Predicting the risk of emergency admission with machine learning: Development and validation using linked electronic health records

**DOI:** 10.1371/journal.pmed.1002695

**Published:** 2018-11-20

**Authors:** Fatemeh Rahimian, Gholamreza Salimi-Khorshidi, Amir H. Payberah, Jenny Tran, Roberto Ayala Solares, Francesca Raimondi, Milad Nazarzadeh, Dexter Canoy, Kazem Rahimi

**Affiliations:** 1 Deep Medicine, Oxford Martin School, Oxford, United Kingdom; 2 The George Institute for Global Health, University of Oxford, Oxford, United Kingdom; 3 Oxford University Hospitals NHS Foundation Trust, Oxford, United Kingdom; Edinburgh University, UNITED KINGDOM

## Abstract

**Background:**

Emergency admissions are a major source of healthcare spending. We aimed to derive, validate, and compare conventional and machine learning models for prediction of the first emergency admission. Machine learning methods are capable of capturing complex interactions that are likely to be present when predicting less specific outcomes, such as this one.

**Methods and findings:**

We used longitudinal data from linked electronic health records of 4.6 million patients aged 18–100 years from 389 practices across England between 1985 to 2015. The population was divided into a derivation cohort (80%, 3.75 million patients from 300 general practices) and a validation cohort (20%, 0.88 million patients from 89 general practices) from geographically distinct regions with different risk levels. We first replicated a previously reported Cox proportional hazards (CPH) model for prediction of the risk of the first emergency admission up to 24 months after baseline. This reference model was then compared with 2 machine learning models, random forest (RF) and gradient boosting classifier (GBC). The initial set of predictors for all models included 43 variables, including patient demographics, lifestyle factors, laboratory tests, currently prescribed medications, selected morbidities, and previous emergency admissions. We then added 13 more variables (marital status, prior general practice visits, and 11 additional morbidities), and also enriched all variables by incorporating temporal information whenever possible (e.g., time since first diagnosis). We also varied the prediction windows to 12, 36, 48, and 60 months after baseline and compared model performances. For internal validation, we used 5-fold cross-validation. When the initial set of variables was used, GBC outperformed RF and CPH, with an area under the receiver operating characteristic curve (AUC) of 0.779 (95% CI 0.777, 0.781), compared to 0.752 (95% CI 0.751, 0.753) and 0.740 (95% CI 0.739, 0.741), respectively. In external validation, we observed an AUC of 0.796, 0.736, and 0.736 for GBC, RF, and CPH, respectively. The addition of temporal information improved AUC across all models. In internal validation, the AUC rose to 0.848 (95% CI 0.847, 0.849), 0.825 (95% CI 0.824, 0.826), and 0.805 (95% CI 0.804, 0.806) for GBC, RF, and CPH, respectively, while the AUC in external validation rose to 0.826, 0.810, and 0.788, respectively. This enhancement also resulted in robust predictions for longer time horizons, with AUC values remaining at similar levels across all models. Overall, compared to the baseline reference CPH model, the final GBC model showed a 10.8% higher AUC (0.848 compared to 0.740) for prediction of risk of emergency admission within 24 months. GBC also showed the best calibration throughout the risk spectrum. Despite the wide range of variables included in models, our study was still limited by the number of variables included; inclusion of more variables could have further improved model performances.

**Conclusions:**

The use of machine learning and addition of temporal information led to substantially improved discrimination and calibration for predicting the risk of emergency admission. Model performance remained stable across a range of prediction time windows and when externally validated. These findings support the potential of incorporating machine learning models into electronic health records to inform care and service planning.

## Abstract 2

With the aid of machine learning, Fatemeh Rahimian and colleagues utilise 25 years’ health data from across England to improve population estimates of the risk of emergency hospital admissions.

## Author summary

**Why was this study done?:**

- We wanted to compare machine learning models to conventional statistical models that are used for risk prediction.
- We also aimed to unlock the power of embedded knowledge in large-scale electronic health records.
- Ultimately, we wanted to provide a tool to help healthcare practitioners accurately predict whether a patient may require emergency care in the near future.

**What did the researchers do and find?:**

- We developed several models for predicting the risk of first emergency admission: 1 Cox proportional hazards (CPH) model and 2 machine learning models. We tested various sets of variables in all models. While the interactions in the CPH model were explicitly specified, the machine learning models benefited from an automatic discovery of the variable interdependencies.
- We validated all models (internally and externally) on a large cohort of electronic health records for over 4.6 million patients from 389 practices across England. For internal validation we used 80% of the data (3.75 million patients from 300 general practices) and performed a 5-fold cross-validation. For external validation we used the remaining 20% of the data (0.88 million patients from 89 general practices) from geographically distinct regions with different risk levels.
- We found that the use of machine learning methods and the addition of more variables and temporal information led to substantially improved discrimination and calibration for predicting the risk of emergency hospital admission. Also machine learning model performance remained stable across a range of prediction time windows and when externally validated.

**What do these findings mean?:**

- The improved and more robust prediction of the risk of emergency hospital admission as a result of the combination of more variables, information about their timing, and better models could help guide policy and practice to reduce the burden of unscheduled admissions.
- By deploying such models in practices, physicians would be able to accurately monitor the risk score of their patients and take the necessary actions in time to avoid unplanned admissions.

## Introduction

Emergency hospital admissions are a major source of healthcare spending [1,2]. In the UK, there were over 5.9 million recorded emergency hospital admissions in 2017, an increase of 2.6% compared to the preceding year [3]. Given the avoidable nature of a large proportion of such admissions, there has been a growing research and policy interest in effective ways of averting them. To guide decision-making, several risk prediction models have been reported [4–9]. However, on average, models tend to have a poor ability to discriminate risk [1,2,10,11]. This might be in part due to limited access to or use of information about risk predictors and their timing, in particular when models use hospital admissions data only. In addition, the relatively non-specific nature of unscheduled hospital admissions—which are often a consequence of a range of health problems as well as provider preferences—suggests the presence of complex relationships between predictors and outcome, which conventional statistical methods are limited at capturing.

The growing availability of comprehensive clinical datasets, such as linked electronic health records (EHRs) with rich information from millions of individuals, together with advances in machine learning offer new opportunities for development of novel risk prediction models that are better at predicting risk. Such models have been shown to outperform standard statistical models, particularly, in settings where clinical data have been richer, and relationships more complex [12–14].

Building on earlier studies and emerging analytical opportunities, we aimed to assess whether application of 2 standard machine learning techniques could enhance the prediction of emergency hospital admissions in the general population compared with a high-performing Cox proportional hazards (CPH) model that also used large-scale EHRs. [8] To better understand when and how machine learning models might achieve a higher performance, we aimed to develop and compare a series of models. In the first step, we used the same set of variables and prediction window (24 months after baseline) as in the previous CPH model. In the next steps, we added more variables and included information about variable timing to the models. To further test the hypothesis that the predictive ability of machine learning models is stronger than that of conventional models when outcomes in the more distant future are to be predicted (because of their ability to better capture multiple known and unknown interactions), we further changed the time horizon for risk prediction to shorter and longer periods.

## Methods

### Study design, data source, and patient selection

The study was conducted using linked EHRs from the UK Clinical Practice Research Datalink (CPRD) study since its inception in 1 January 1985 to 30 September 2015 (https://www.cprd.com) [15]. The CPRD database is pseudo-anonymised patient data from 674 general practices in the UK, covering approximately 7% of the current UK population, and is broadly representative of the UK population by age, sex, and ethnicity. It links primary care records with discharge diagnoses from Hospital Episode Statistics [16] and mortality data from national death registries (Office for National Statistics) with a coding system equivalent to the World Health Organization International Classification of Diseases–10th Revision (ICD-10) [17]. The CPRD dataset is one of the most comprehensive prospective primary care databases, the validity of which has previously been reviewed elsewhere [18,19]. In this study, a subset of the CPRD dataset—which covers different regions of England—was used. The scientific approval for this study (protocol no: 17_224R2) was given by the CPRD Independent Scientific Advisory Committee, and no additional informed consent was required as there was no individual patient involvement [20].

We considered all patients aged 18 to 100 years with at least 1 year of registration with a general practice in this study, and excluded those without a valid National Health Service (NHS) number or missing information on Index of Multiple Deprivation (IMD), an area-based socioeconomic status indicator, with increasing level of deprivation with higher scores [21]. Similar to our benchmark CPH model (QAdmissions) [8], the entry date to the study for each patient was defined as the latest of their 18th birthday or date of first registration with a practice plus 1 year, provided that this date is before the baseline (1 January 2010). From the total of 7,612,760 patients in the database, 4,637,297 patients met these selection criteria. On average we had 405 months of recorded data per patient (minimum 5, maximum 977, standard deviation 235, median 362), which summed up to a total of over 1.86 billion patient-months of data. We censored patients at the earliest date of first emergency hospital admission, death, transfer out of practice, or end of the study.

### Predictors

We used 3 different sets of predictors (variables used in the prediction models). In the first set of predictors, referred to as QA, we included 43 variables from the established QAdmissions model [8], covering patient demographics (age, sex, and ethnicity), lifestyle factors (socioeconomic status, body mass index [BMI], smoking status, and alcohol consumption), strategic health authority (SHA) (region), family history of chronic disease, various laboratory tests, 16 comorbidities, 6 prescribed medications, and previous emergency admissions. In the second set of predictors, referred to as QA+, we extended QA by adding 13 new predictors, including marital status, 11 new comorbidities, and the number of general practice visits in the year before baseline. In the third set of predictors, referred to as T (for temporal), we modified some of the QA+ predictors to hold temporal information: instead of a binary variable for diagnosis of comorbidities, we considered the time since first recorded diagnosis; instead of previous utilisation of healthcare service, we considered time since last use of healthcare service; and instead of a binary value for laboratory tests being recorded, we considered time since the latest laboratory tests. Table 1 shows the complete list of predictors, and how these predictors have been represented in each of our variable sets.

**Table 1:** Predictors considered, and how they are represented in CPRD and in our models.

Category	Predictor	Representation in CPRD	Representation in models	In QA	In QA+	In T
**Demographics**	Age	Year of birth	Computed based on mid-year of year of birth	*	*	*
	Sex	Binary variable (male/female)	Binary variable	*	*	*
	Ethnicity	Ethnicity (categorical value)	Categorical variable	*	*	*
**Lifestyle and family history**	Socioeconomic status	Index of Multiple Deprivation	Numeric variable on a scale of 1 to 5	*	*	*
	BMI	Weight measurement recorded repeatedly in various clinic visits	BMI based on height and most recent recorded weight	*	*	*
	Smoking status	Current tobacco use, in terms of number of cigars/cigarettes per day (recorded repeatedly)	Categorical variable for latest status: non-smoker, ex-smoker, light smoker (less than 10 cigarettes/day), moderate smoker (10–20 cigarettes/day), heavy smoker (more than 20 cigarettes/day), smoker (amount not recorded)	*	*	*
	Alcohol intake	Current alcohol consumption, in terms of units of alcohol per day (recorded repeatedly)	Categorical variable for latest status: non-drinker, ex-drinker, trivial (less than 1 unit/week), light (1–2 units/week), moderate (3–6 units/week), heavy (7–9 units/week), very heavy (more than 9 units/week), drinker (amount not recorded)	*	*	*
	Family history of chronic disease	Binary variable (yes/no)	Binary variable (yes/no)	*	*	*
	Strategic health authority (region)	Categorical variable	Categorical variable	*	*	*
	Marital status	Categorical variable	Categorical variable		*	*
**Use of care**	Previous emergency admissions	Read Code and date of event	Number of occurrences during last year	*	*	*
			Time since last occurrence (in days)			*
	Prior GP visits (consultations)	Read Code and date of event	Number of occurrences during last year		*	*
			Time since last occurrence (in days)			*
			Total duration spent in GP visits (minutes)			*
**Clinical diagnoses (comorbidities)**	Diabetes, atrial fibrillation, cardiovascular disease, congestive cardiac failure, venous thromboembolism, cancer, asthma or COPD, epilepsy, falls, manic depression or schizophrenia, chronic renal disease, chronic liver disease or pancreatitis, valvular heart disease, treated hypertension, rheumatoid arthritis or SLE, depression (QOF definition)	Read Code and date of entry	One separate binary variable for each disease, 16 variables in total	*	*	
			Time since first diagnosis (in days)—1 separate variable for each disease, 16 variables in total			*
	Arthritis, connective tissue disease, hemiplegia, HIV/AIDS, hyperlipidaemia, learning disability, obesity, osteoporosis, peripheral arterial disease, peptic ulcer disease, substance abuse	Read Code and date of entry	One separate binary variable for each disease, 11 variables in total		*	
			Time since first diagnosis (in days)—1 separate variable for each disease, 11 variables in total			*
**Clinical measures and laboratory tests**	Systolic blood pressure, haemoglobin, cholesterol/HDL, liver function test (γ-GT, aspartate aminotransferase, or bilirubin), platelets, ESR	Numeric value for result and date of measurement	Binary (yes/no) variable for if recorded—1 variable per test	*	*	
			Numeric variable for most recent result—1 variable per test	*	*	*
			Binary variable for abnormal result—1 variable per test	*	*	*
			Time since the latest result (in days)—1 variable per test			*
**Prescriptions**	Statin, NSAID, anticoagulant, corticosteroid, antidepressant, antipsychotic	Date of prescription if applicable	Binary (yes/no) variable for if prescription exists	*	*	*

γ-GT, γ-glutamyl transferase; COPD, chronic obstructive pulmonary disease; CPRD, Clinical Practice Research Datalink; ESR, erythrocyte sedimentation rate; GP, general practice; HDL, high-density lipoprotein; NSAID, non-steroidal anti-inflammatory drug; QOF, Quality and Outcomes Framework; SLE, systemic lupus erythematosus.

Data missingness for each variable can be found in Table 2. For laboratory tests and clinical measurements, we marked the missingness as a binary variable (recorded/not recorded). Clinical diagnoses (morbidities) were assumed to be present only if they had been recorded. Missing data for BMI (29%), smoking status (17%), and alcohol intake (29%) were imputed using multiple imputation with chained equations [22–24] and combined using Rubin’s rule. It is important to note that for these 3 variables, data are not missing at random. Therefore, there is always a risk of bias when imputation is employed. However, this limitation tends to be largely relevant to epidemiological studies that seek to identify specific risk factors rather than risk prediction overall. Nevertheless, we ran the models with and without imputation and observed that the differences in outcomes between models remain pretty much constant. These results are reported in S1 Table. Indeed, the fact that the imputed data have not led to substantial bias in our study can be directly assessed with the calibration plots, which show a good match between the predicted and actual probabilities of outcomes.

**Table 2:** Baseline characteristics of derivation and validation cohorts.

Predictor		Derivation cohort *n* = 3,749,932	Validation cohort *n* = 887,365
**Sex, *n* (%)**			
	Female	1,937,265 (51.66)	454,424 (51.21)
	Male	1,812,667 (48.34)	432,941 (48.79)
**Age, mean (SD)**		51.0 (19.8)	53.1 (19.9)
**Marital status, *n* (%)**	Missing	2,960,949 (78.96)	735,198 (82.85)
	Single	481,753 (12.85)	44,941 (5.06)
	Married/stable relationship	481,753 (12.85)	94,000 (10.60)
	Separated/widowed	64,441 (1.72)	13,226 (1.49)
**IMD score (socioeconomic status), mean (SD)**		2.8 (1.4)	3.3 (1.4)
**Family history of chronic disease, *n* (%)**		646,360 (17.24)	196,800 (22.18)
**BMI**	Missing, *n* (%)	1,094,892 (29.20)	242,324 (27.31)
	Mean (SD)	26.1 (5.6)	26.4 (5.8)
**Strategic health authority (region), *n* (%)**			
	North East	0	89,004 (10.03)
	North West	0	613,460 (69.13)
	Yorkshire and the Humber	0	184,901 (20.84)
	East Midlands	150,831 (4.02)	0
	West Midlands	518,586 (13.83)	0
	East of England	540,346 (14.41)	0
	South West	558,036 (14.88)	0
	South Central	572,791 (15.27)	0
	London	817,870 (21.81)	0
	South East Coast	591,472 (15.77)	0
**Ethnicity, *n* (%)**			
	Missing	2,625,523 (70.02)	536,806 (60.49)
	White	1,039,476 (27.72)	339,466 (38.26)
	Indian	16,740 (0.45)	1,571 (0.18)
	Pakistani	6,153 (0.16)	2,395 (0.27)
	Bangladeshi	1,958 (0.05)	355 (0.04)
	Other Asian	7,466 (0.20)	711 (0.08)
	Caribbean	9,786 (0.26)	541 (0.06)
	Black African	15,499 (0.41)	1,234 (0.14)
	Chinese	3,493 (0.09)	810 (0.09)
	Other	23,838 (0.64)	3,476 (0.39)
**Smoking status, *n* (%)**			
	Missing	680,838 (18.16)	143,032 (16.12)
	Non-smoker	1,678,287 (44.76)	379,395 (42.76)
	Ex-smoker	392,806 (10.48)	90,539 (10.20)
	Light smoker (<10 cigarettes/day)	286,113 (7.63)	69,704 (7.86)
	Moderate smoker (10–20 cigarettes/day)	345,639 (9.22)	102,533 (11.55)
	Heavy smoker (>20 cigarettes/day)	250,567 (6.68)	80,713 (9.10)
	Smoker, amount not recorded	115,367 (3.08)	21,321 (2.40)
**Alcohol intake, *n* (%)**			
	Missing	1,089,383 (29.05)	235,862 (26.58)
	Non-drinker	360,048 (9.60)	82,905 (9.34)
	Ex-drinker	24,802 (0.66)	8,339 (0.94)
	Trivial (<1 unit/week)	249,020 (6.64)	46,001 (5.18)
	Light (1–2 units/week)	447,954 (11.95)	99,739 (11.24)
	Moderate (3–6 units/week)	418,400 (11.16)	101,713 (11.46)
	Heavy (7–9 units/week)	161,290 (4.30)	40,942 (4.61)
	Very heavy (>9 units/week)	627,261 (16.73)	194,999 (21.98)
	Drinker, amount not recorded	371,774 (9.91)	76,865 (8.66)
**Previous use of healthcare service**			
	No emergency admission, *n* (%)	3,583,848 (95.57)	834,693 (94.06)
	1 emergency admission, *n* (%)	120,614 (3.22)	36,046 (4.06)
	2 emergency admissions, *n* (%)	30,111 (0.80)	10,546 (1.19)
	3+ emergency admissions, *n* (%)	15,359 (0.41)	6,080 (0.69)
	Mean number of days since last admission (SD)	170.7 (103.7)	169.6 (105.8)
	Mean number of consultations (SD)	21.7 (24.4)	24.4 (25.9)
	Mean consultation duration	124.9 (227.5)	163.0 (374.3)
	Mean number of days since last consultation (SD)	300.8 (83.4)	307.4 (78.7)
**Clinical values**			
Systolic blood pressure	Missing, *n* (%)	443,729 (11.83)	98,150 (11.06)
	Mean (SD)	127.9 (18.4)	128.6 (19.2)
Cholesterol/HDL	Missing, *n* (%)	2,781,874 (74.18)	593,814 (66.92)
	Mean (SD)	3.8 (1.6)	3.8 (1.8)
Haemoglobin	Missing, *n* (%)	2,012,077 (53.66)	4434,005 (48.91)
	Haemoglobin < 110 g/l, *n* (%)	84,396 (2.25)	23,178 (2.61)
Platelets	Missing, *n* (%)	12,056,437 (54.84)	449,522 (50.66)
	Platelets > 480 × 10^9^/l, *n* (%)	21,305 (0.57)	5,900 (0.66)
Liver function test	Missing, *n* (%)	2,285,715 (60.95)	489,673 (55.18)
	Abnormal liver function test, *n* (%)	23,217 (0.62)	9,328 (1.05)
ESR	Missing, *n* (%)	2,908,165 (77.55)	683,599 (77.04)
	Abnormal ESR, *n* (%)	96,436 (2.57)	21,828 (2.46)
**Comorbidity, *n* (%)**			
	Diabetes	326,672 (8.71)	83,309 (9.39)
	Atrial fibrillation	122,627 (3.27)	49,647 (5.59)
	Cardiovascular disease	379,071 (10.11)	104,215 (11.74)
	Congestive cardiac failure	140,439 (3.75)	53,742 (6.06)
	Venous thromboembolism	99,083 (2.64)	36,791 (4.15)
	Cancer	143,923 (3.84)	36,677 (4.13)
	Asthma or COPD	753,223 (20.09)	162,853 (18.35)
	Epilepsy	103,800 (2.77)	7,690 (0.87)
	Falls	354,748 (9.46)	86,801 (9.78)
	Manic depression or schizophrenia	33,716 (0.90)	0 (0.00)
	Chronic renal disease	272,292 (7.26)	72,221 (8.14)
	Chronic liver disease or pancreatitis	68,726 (1.83)	0 (0.00)
	Valvular heart disease	49,274 (1.31)	0 (0.00)
	Treated hypertension	892,430 (23.8)	193,826 (21.84)
	Rheumatoid arthritis or SLE	58,658 (1.56)	0 (0.00)
	Depression (QOF definition)	862,357 (23.0)	173,965 (19.6)
	Arthritis	52,4936 (14.0)	161,050 (18.15)
	Connective tissue disease	32,850 (0.88)	7,079 (0.80)
	Hemiplegia	7,097 (0.19)	2,553 (0.29)
	HIV/AIDS	29,701 (0.79)	7,176 (0.81)
	Hyperlipidaemia	216,304 (5.77)	66,238 (7.46)
	Learning disability	18,574 (0.50)	5,069 (0.57)
	Obesity	231,123 (6.16)	66,210 (7.46)
	Osteoporosis	66,877 (1.78)	20,056 (2.26)
	Peripheral arterial disease	56,828 (1.52)	20,761 (2.34)
	Peptic ulcer disease	62,122 (1.66)	24,151 (2.72)
	Substance abuse	54,517 (1.45)	19,673 (2.22)
**Current prescribed medication, *n* (%)**			
	Statin	552,982 (14.75)	164,814 (18.57)
	NSAID	1505,161 (40.14)	423,637 (47.74)
	Anticoagulant	122,803 (3.27)	34,285 (3.86)
	Corticosteroid	809,336 (21.58)	214,067 (24.12)
	Antidepressant	649,131 (17.31)	210,259 (23.69)
	Antipsychotic	114,487 (3.05)	40,060 (4.51)

COPD, chronic obstructive pulmonary disease; ESR, erythrocyte sedimentation rate; HDL, high-density lipoprotein; IMD, Index of Multiple Deprivation; NSAID, non-steroidal anti-inflammatory drug; QOF, Quality and Outcomes Framework; SLE, systemic lupus erythematosus.

Finally, we ended up with 58, 80, and 121 variables for the QA, QA+, and T predictor sets, respectively.

### Outcome and time windows

The outcome of interest was the first emergency admission to hospital after baseline (1 January 2010), as recorded by the general practice, using the Read Codes shown in S2 Table. The QAdmissions model reported model performance for outcomes occurring within a 24-month time window after baseline. In our primary analyses we chose the same time window, but to further assess model stability for predicting outcomes during different time frames, we varied the prediction window to shorter (12 months) and longer periods (36, 48, and 60 months) after baseline. Unless stated otherwise, where we refer to the outcome, we consider the 24-month time window.

### Derivation and validation of models

We first replicated the CPH model as the benchmark model [8]. This model was based on the same predictor variables in [8] and included all the interaction and fractional polynomial terms as previously reported. Since QAdmissions reported results separately for men and women, we also analysed the results stratified by sex. However, in the absence of any material difference by sex, and for brevity, we combined data for men and women in subsequent analyses.

We compared the CPH model to 2 machine learning models, namely gradient boosting classifier (GBC) [25] and random forest (RF) [26]. Both GBC and RF models were used as ensemble models based on decision trees [27], but each represented a distinct family of ensemble learning methods [28]—boosting [29,30] and bagging [31], respectively. Boosting refers to any ensemble method that can combine several weak learners into a strong learner. The general idea of most boosting methods is to train predictors sequentially, each trying to correct its predecessor. By contrast, bagging uses the same training algorithm multiple times in parallel (e.g., a RF employs multiple decision trees), but trains them on different random subsets of the data. When sampling is performed with replacement, this method is called bagging. These 2 models were chosen because they are shown to outperform other machine learning models on a variety of datasets, are fairly robust and applicable to big datasets, and require little modification of parameters prior to modelling [32]. These machine learning methods work on both categorical and numerical variables in any scale, obviating the need for conversion of features or normalisation of their values. We tuned the hyperparameters of GBC and RF after a broad search of parameter space. For brevity, we only report the results with the selected values for the parameters, which are listed in S3 Table.

To assess the impact of more variables and their timing, all 3 models were repeated using the extended QA+ and T variables [33]. Consequently, we ended up with 9 different models (3 sets of predictors and 3 modelling techniques) that were compared against each other. In the final step, we studied the performance of all 9 models for predicting events over shorter and longer time windows than the original 24-month window.

### Evaluation methodology and metrics

To measure the performance of models, both internal and external validation were used. For external validation, we followed the recommendations of [34] and chose a non-random subset of data from different SHAs, the regional health commissioning bodies of the NHS. More precisely, we selected 3 SHAs (North East, North West, and Yorkshire and the Humber) that had different statistical properties in terms of socioeconomic status and the rate of first emergency admission as our validation cohort (20% of the total population). We argue that our selection of 3 SHAs with different statistical characteristics is similar to using an external dataset, and provides, therefore, a better case for showing whether or not the model is overfitting. This, however, inevitably means that certain features are not available in the validation cohort. By taking this approach, we consciously sacrificed model optimisation to assess any possible model overfitting (and hence poor external validity). For internal validation, we used the remaining SHAs (80% of the population), and performed a 5-fold cross-validation [35–37].

While our outcome is binary, instead of just predicting 0 or 1 for a patient (being admitted or not), we predicted the probability of that patient belonging to class 1 (being admitted). This enabled us to measure the area under the receiver operating characteristic curve (AUC), a discrimination metric, which is equal to the probability that a classifier will rank a randomly chosen positive instance higher than a randomly chosen negative one. Model calibration was assessed with calibration curves [38], where for a perfectly calibrated model the curve is mapped to the identity line (*y* = *x*). To this end, the prediction space is discretized into 10 bins. Cases with predicted value in the range [0, 0.1) fall in the first bin, values in the range [0.1, 0.2) in the second bin, etc. For each bin, the mean predicted value is plotted against the true fraction of positive cases. The reported results for AUC and calibration demonstrate the average of these values over 5 different folds. The standard deviation across the folds is marked by a shaded area around the average.

Discrimination and calibration metrics were supplemented with positive and negative predictive values, as well as precision and recall for all models. Finally, to assess the possibility of bias in estimates, we stratified AUC by practice and present the findings in a funnel plot by practice-level rate of emergency admissions [39].

We report our findings in accordance to the Guidelines for Developing and Reporting Machine Learning Predictive Models in Biomedical Research [40] and Transparent Reporting of a Multivariable Prediction Model for Individual Prognosis or Diagnosis (TRIPOD) [41]. We performed all statistical analyses using Python version 2.7 and R version 3.3.

## Results

### Basic statistics

The baseline characteristics of the derivation and validation cohorts are shown in Table 2. Expectedly, the cohorts differed in some respects, such as age and ethnicity. The proportions of women and men in the derivation and validation cohorts were comparable, but the derivation cohort had a slightly higher mean IMD than the validation cohort (2.8 versus 3.3).

Hospital admission rates were on average 7.8% for the regions in the derivation cohort. The admission rates were slightly higher in the validation cohort overall, across all age groups (except the oldest age group) and between sexes. The North East and North West regions had the highest admission rates, which were 12% and 11%, respectively. Yorkshire and the Humber had a rate of 6%, which brought the average rate of outcome to 10.4% in the validation cohort. The rates of the first emergency admission within 2 years, according to age, sex and SHA, are provided in S4 Table. Also, the rate of outcome by duration of follow-up, presented in S5 Table, demonstrates increasing rate with longer duration of follow-up.

### Model performance

Using QA predictors in the CPH model, AUC was 0.740 (0.741 for men and 0.739 for women). Applying RF and GBC to the same predictors increased the AUC to 0.752 and 0.779, respectively (Table 3). Using QA+ predictors, all models showed a slightly higher AUC, with the largest increase seen for the RF model. When T predictors were used, all models showed higher AUC values, but again the GBC model showed the best performance (0.805, 0.825, and 0.848 for the CPH, RF, and GBC models, respectively). The corresponding receiver operating characteristic (ROC) curves for these models are presented in S1 Fig.

**Table 3:** Cross-validated model discrimination for different predictor sets and modelling techniques: Derivation cohort.

Predictor set	Model					
	CPH		RF		GBC	
	AUC	95% CI	AUC	95% CI	AUC	95% CI
**QA (men only)**	0.741	0.739, 0.743	0.754	0.752, 0.756	0.777	0.775, 0.779
**QA (women only)**	0.739	0.738, 0.740	0.755	0.754, 0.756	0.779	0.777, 0.781
**QA**	0.740	0.739, 0.741	0.752	0.751, 0.753	0.779	0.777, 0.781
**QA+**	0.751	0.750, 0.753	0.822	0.818, 0.826	0.834	0.833, 0.835
**T**	0.805	0.804, 0.806	0.825	0.824, 0.826	0.848	0.847, 0.849

For any given set of predictors, GBC outperforms the other 2 models. Similarly, for any given model, T predictors show the best predictive power.

AUC, area under the receiver operating characteristic curve; CPH, Cox proportional hazards; GBC, gradient boosting classifier; RF, random forest.

**Fig 1:**
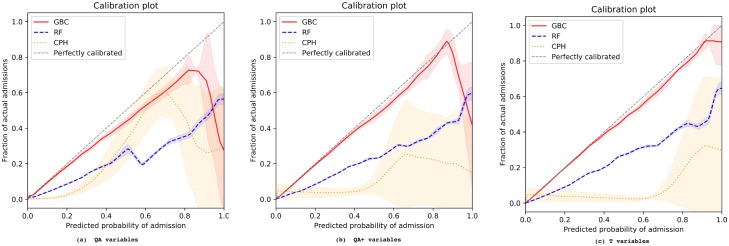
Cross-validated model calibration for different predictor sets and modelling techniques. (a) QA variables; (b) QA+ variables; (c) T variables. The *x*-axis shows the predicted probability of emergency admission, while the *y*-axis shows the fraction of actual admissions for each predicted probability. The shaded areas depict the standard deviation across different folds in a 5-fold cross-validation. CPH, Cox proportional hazards; GBC, gradient boosting classifier; RF, random forest.

Fig 1 illustrates the calibration of all models stratified by predictor set. GBC constantly exhibited the best calibration across all settings. Using QA+ and T predictors improved the calibration of RF and GBC models, compared to using QA predictors. However, the opposite was the case when the extended set of variables was applied to the CPH model: its calibration degraded with the addition of new variables. Analysis of positive and negative predictive values, precision, and recall as complementary metrics (S2–S4 Figs) supported the main findings of a higher performance of the GBC model, in particular together with T predictors.

### Variable importance

In the GBC and RF models, the relative importance of predictors is readily identifiable through ranking of their repeated selection across multiple trees and higher up the trees. Making use of this ranking, we report the top variables among the QA, QA+, and T sets in S6 and S7 Tables. These tables show that, for example, when GBC is used together with the QA set, age, laboratory test results (such as cholesterol ratio, haemoglobin, and platelets), systolic blood pressure, and the number of admissions during the last year are among the top predictors. With the QA+ set, GBC ranks the number of previous consultations during the last year as the most important predictor, followed by age and not only the laboratory test results, but also the frequency of those tests being reported. Finally, when T variables are used, both the number and the duration of consultations are shown to be highly predictive, together with age and time since last admission and consultation, while laboratory test results remain among the top predictors.

### External validation

**Fig 2:**
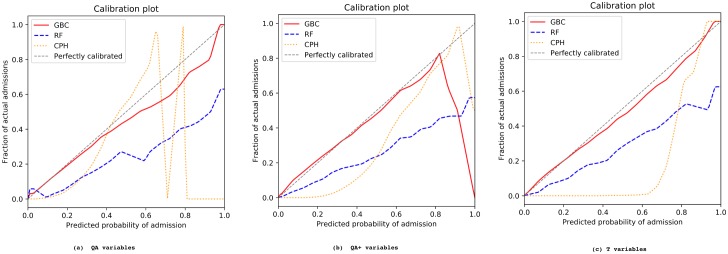
Externally validated model calibration for different predictor sets and modelling techniques. (a) QA variables; (b) QA+ variables; (c) T variables. The *x*-axis shows the predicted probability of emergency admission, while the *y*-axis shows the fraction of actual admissions for each predicted probability. CPH, Cox proportional hazards; GBC, gradient boosting classifier; RF, random forest.

The AUC values from the external validation cohort are reported in Table 4, and their corresponding ROC curves are shown in S5 Fig. All models showed a slight decline in AUC compared to internally validated AUC. Note that, while region is one of the input variables for training the models, this information is missing when external validation is performed because the regions in the validation cohort are unknown to the constructed models. Yet, the decline in AUC was, on average, less than 1% for CPH and less than 2% for the RF and GBC models, and our best model (GBC with T predictors) gave an AUC of 0.826 on the validation data. This model also showed an excellent calibration (See Fig 2), and the funnel plot in S6 Fig shows no bias by practice population and admission rate for all practices in both the derivation and validation cohorts. Other metrics and plots, provided in S7–S9 Figs, also demonstrate the consistency between the derivation and validation results.

**Table 4:** Externally validated model discrimination for different predictor sets and modelling techniques: Validation cohort.

Predictor set	Model		
	CPH	RF	GBC
**QA**	0.736	0.736	0.796
**QA+**	0.743	0.799	0.810
**T**	0.788	0.810	0.826

Predictor set T and GBC modelling constantly perform better than their counterparts. The results conform to the pattern observed in internal cross-validation.

CPH, Cox proportional hazards; GBC, gradient boosting classifier; RF, random forest.

### Impact of change in prediction time window

**Fig 3:**
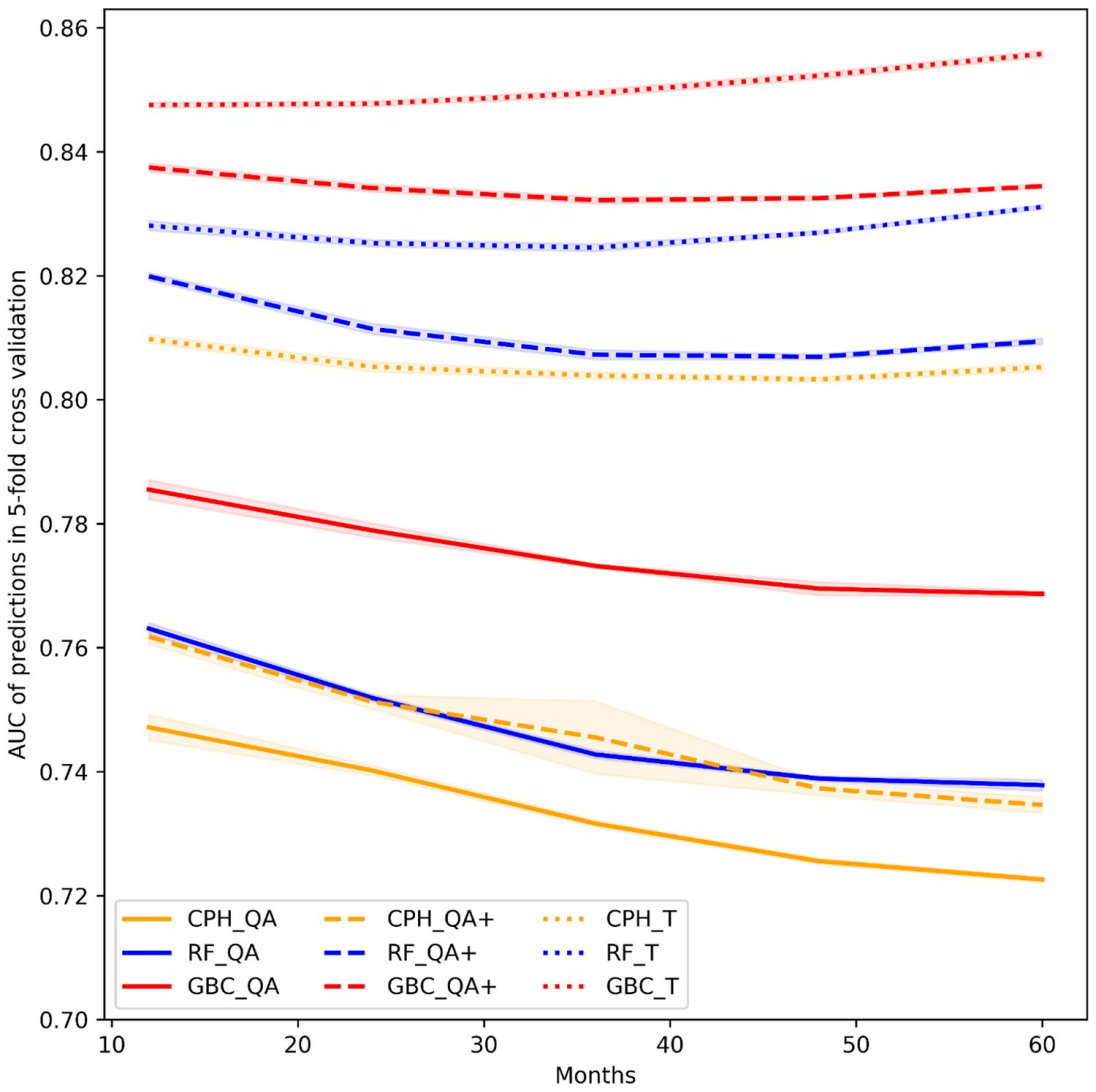
Model discrimination for different follow-up periods (from 12 to 60 months after baseline). Colours differentiate the 3 modelling techniques (GBC, RF, and CPH), whereas line styles indicate the predictor sets (QA, QA+, and T). AUC, area under the receiver operating characteristic curve; CPH, Cox proportional hazards; GBC, gradient boosting classifier; RF, random forest.

Fig 3 illustrates the AUC of all models when predicting the outcome within 1 to 5 years after the baseline. Models using QA and QA+ predictors showed better performance in predicting emergency admissions that occur early than those that occur late during the follow-up period. The CPH, RF, and GBC models all exhibited more stable performance for predicting emergency admissions—both in the shorter and longer follow-up time windows—when using T predictors. These results suggest that variables that capture time since prior events (e.g., time since first diagnosis of a comorbidity or time since last laboratory test) are stronger predictors than their binary counterparts.

## Discussion

We show that our machine learning models have substantially higher performances in predicting the risk of emergency hospital admission than one of the best available statistical models based on routinely collected data from the same setting. Stepwise modification of modelling techniques and variables extracted from EHRs of 4.6 million patients led to an overall improvement in model discrimination from AUC 0.74 to AUC 0.85 in the internal validation cohort and from AUC 0.74 to 0.83 in the external validation cohort. Although the benchmark CPH model also showed an improvement in discrimination properties when additional variables were added, this came at the cost of worsening model calibration, with substantial overestimation of risk across almost all risk levels. By contrast, calibration of the GBC model was further enhanced when additional variables were added. We show that model performance was retained over longer prediction windows (up to 5 years after baseline) when models incorporated additional information about the timing of variables (e.g., time since first diagnosis of a comorbidity or time since last laboratory test), which is typically ignored in traditional models.

A few previous studies have applied machine learning techniques to predict the risk of hospital re-admission [42,43] or frequency of emergency department visits [44] and have reported highly promising findings. However, since these studies used hospital data only, they were unable to assess the risk of first emergency admission in a non-hospitalised population. On the other hand, studies investigating the risk of emergency hospital admission, as in our report, have mainly utilised statistical models [2,4,6–8,10,45]. Although their predictive ability has on average been limited, some of the more complex models have achieved high levels of accuracy in risk prediction. To test whether machine learning models could improve such models, we chose a state-of-the-art statistical model with high performance as our benchmark model [8].

We show that in the presence of large cohorts with rich information about individuals, machine learning models outperform one of the best conventional statistical models by learning from the data, with little requirement for transformation of the predictors or model structure. The stepwise changes to selected variables and their modelling suggested that the better performance observed was likely due to the higher ability of machine learning models to automatically capture and benefit from existing (a priori unknown) interactions and complex non-linear decision boundaries.

Our study findings should be interpreted in light of their strengths and limitations. One of the key strengths of our work is the direct comparison of machine learning models with one of the best statistical models as the benchmark model. The stepwise changes to modelling techniques, predictors, and time windows for risk prediction revealed when and how model performance could be improved. Another strength of our study is its conservative approach of non-random data splitting for external validation. Although this approach has been recommended [34], in particular when EHR data are used, it has not been widely adopted. EHR models typically divide the study database randomly into derivation and validation subsets, which is more prone to model overfitting. Despite our approach, the generalisability of our best performing machine learning model to other settings may be limited and requires further evaluation. The field of machine learning is advancing rapidly. In our study, we employed 2 readily available machine learning models based on their relative flexibility in handling predictors with no need for variable pre-processing. Although this makes the models useful from a practical point of view, we believe that there is still some room for improving risk prediction by adopting other techniques and a wider range of variables. Finally, we chose emergency hospital admission as the outcome assuming that its non-specific and complex nature is more suitable to data-driven machine learning than more specific clinical outcomes such as myocardial infarction, with well-established risk factors and risk markers. Whether machine learning models can lead to similarly strong improvements in risk prediction in other areas of medicine requires further research.

The improved and more robust prediction of the risk of emergency hospital admission as a result of the combination of more variables, information about their timing, and better models could help guide policy and practice to reduce the burden of unscheduled admissions. By deploying such a model in practices, physicians would be able to monitor the risk score of their patients and take the necessary actions in time to avoid unplanned admissions. It is important to note that hospital admissions in general are affected not only by patient profiles, but also by the policies governing care providers regarding whom they admit and what the risk tolerances are. Notwithstanding the further opportunities for improvement, we believe that routine integration of our best performing model into EHRs is both feasible (as is the case for QRISK [46, 47]) and likely to lead to better decision-making for patient screening and proactive care.

## Supporting information

S1 Fig ROC curves for different modelling techniques (GBC, RF, and CPH) with different predictor sets (QA, QA+, and T) in the derivation cohort. GBC constantly shows a better AUC regardless of the predictors used.
(TIF)

S2 Fig Positive predictive value for various thresholds (cohort: derivation). The values are the average across 5 folds.
(TIF)

S3 Fig Negative predictive value for various thresholds (cohort: derivation). The values are the average across 5 folds.
(TIF)

S4 Fig Cross-validated precision and recall for different predictor sets and modelling techniques (cohort: derivation). The values for all the folds are shown. These plots show the trade-off between precision and recall. Within any one model, one can also decide to emphasise either precision or recall.
(DOCX)

S5 Fig ROC curves for different modelling techniques (GBC, RF, and CPH) with different predictor sets (QA, QA+, and T) in the validation cohort. GBC constantly shows a better AUC regardless of the predictors used.
(TIF)

S6 Fig Discriminatory ability of the final model (GBC with T predictors), stratified by practice-level admission rate for both the validation and derivation cohorts. AUC is for the final model (GBC with T predictors). Colours indicate different cohorts, while the size of each bubble reflects the population of the corresponding practice. The solid and dashed lines show the 99.9% and 95% confidence intervals, respectively.
(TIF)

S7 Fig Positive predictive value for various thresholds (cohort: validation). (TIF)

S8 Fig Negative predictive value for various thresholds (cohort: validation). (TIF)

S9 Fig Externally validated precision and recall for different predictor sets and modelling techniques. These plots show the trade-off between precision and recall. Within any one model, one can also decide to emphasise either precision or recall.
(DOCX)

S1 Table AUC of the models with 3 different imputation models: (1) without imputation, dropping rows with null value for BMI, smoking, and alcohol; (2) without imputation, replacing the null values for BMI, smoking, and alcohol with a default out-of-range value, e.g., −1; and (3) with imputation (multiple imputation with chained equations). (DOCX)

S2 Table Med codes and Read Codes for identification of emergency hospital admissions. (DOCX)

S3 Table Hyperparameters that are used in the machine learning models. (DOCX)

S4 Table Rate of first emergency hospital admission in 2 years overall and stratified by sex, age, and SHA in the derivation and validation cohorts. (DOCX)

S5 Table Rate of first emergency hospital admission, by duration of follow-up. (DOCX)

S6 Table Top 20 strongest predictors of QA, QA+, and T, when GBC is used. (DOCX)

S7 Table Top 20 strongest predictors of QA, QA+, and T, when RF is used. (DOCX)

## Abbreviations

AUC: area under the receiver operating characteristic curve
BMI: body mass index
CPH: Cox proportional hazards
CPRD: Clinical Practice Research Datalink
EHR: electronic health record
GBC: gradient boosting classifier
IMD: Index of Multiple Deprivation
NHS: National Health Service
RF: random forest
ROC: receiver operating characteristic
SHA: strategic health authority
