# Supplementary material for: Predicting the risk of emergency admission with machine learning: Development and validation using linked electronic health records
Source: PLoS Med. 2018 Nov 20;15(11):e1002695. doi: 10.1371/journal.pmed.1002695 (PMC6245681; doi:10.1371/journal.pmed.1002695)
Supplement: S4 Fig — The values for all the folds are shown. These plots show the trade-off between precision and recall. Within any one model, one can also decide to emphasise either precision or recall. (DOCX) [file pmed.1002695.s004.docx]

| Model | QA | QA+ | T |
| --- | --- | --- | --- |
| CPH | 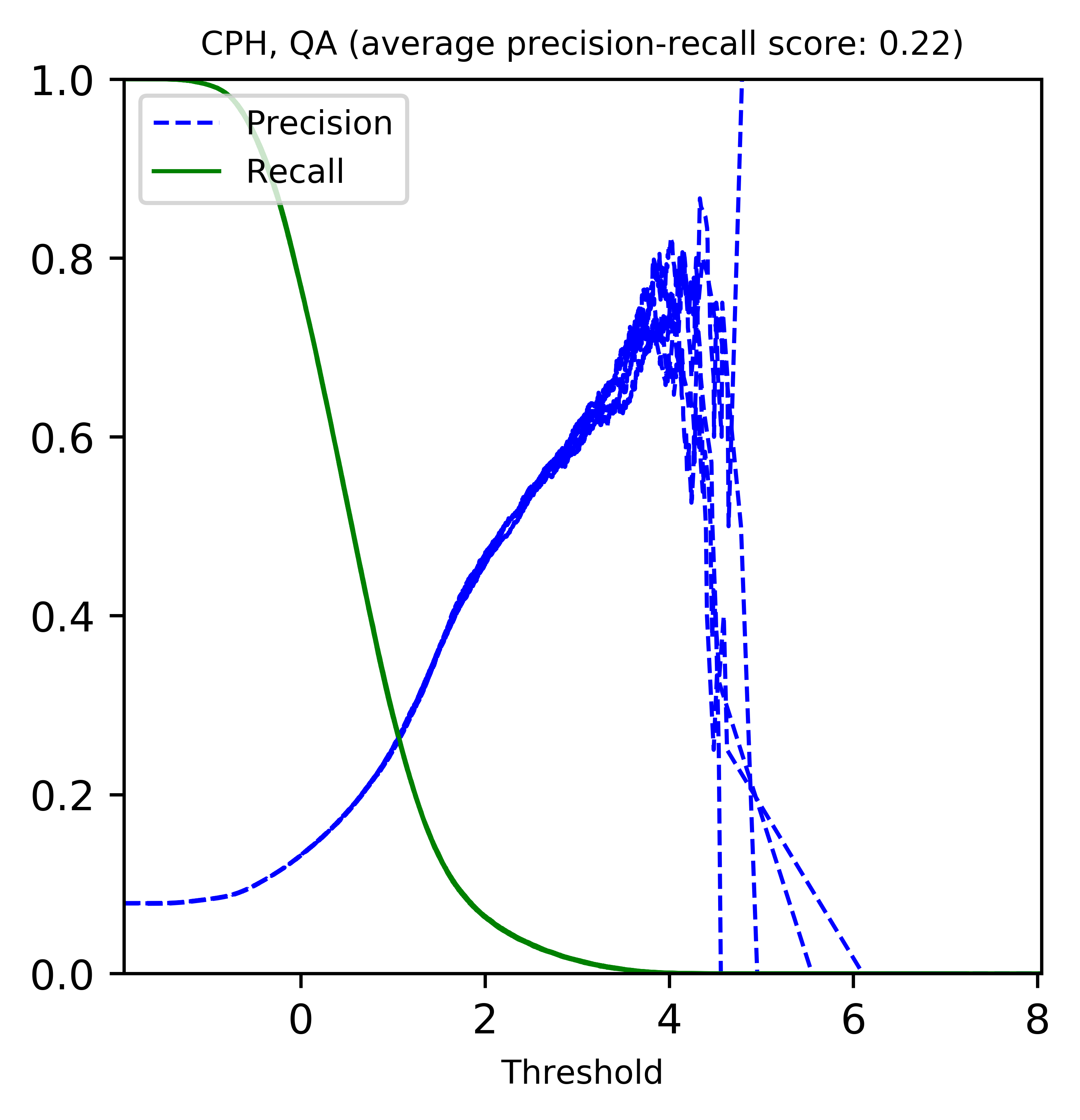 | 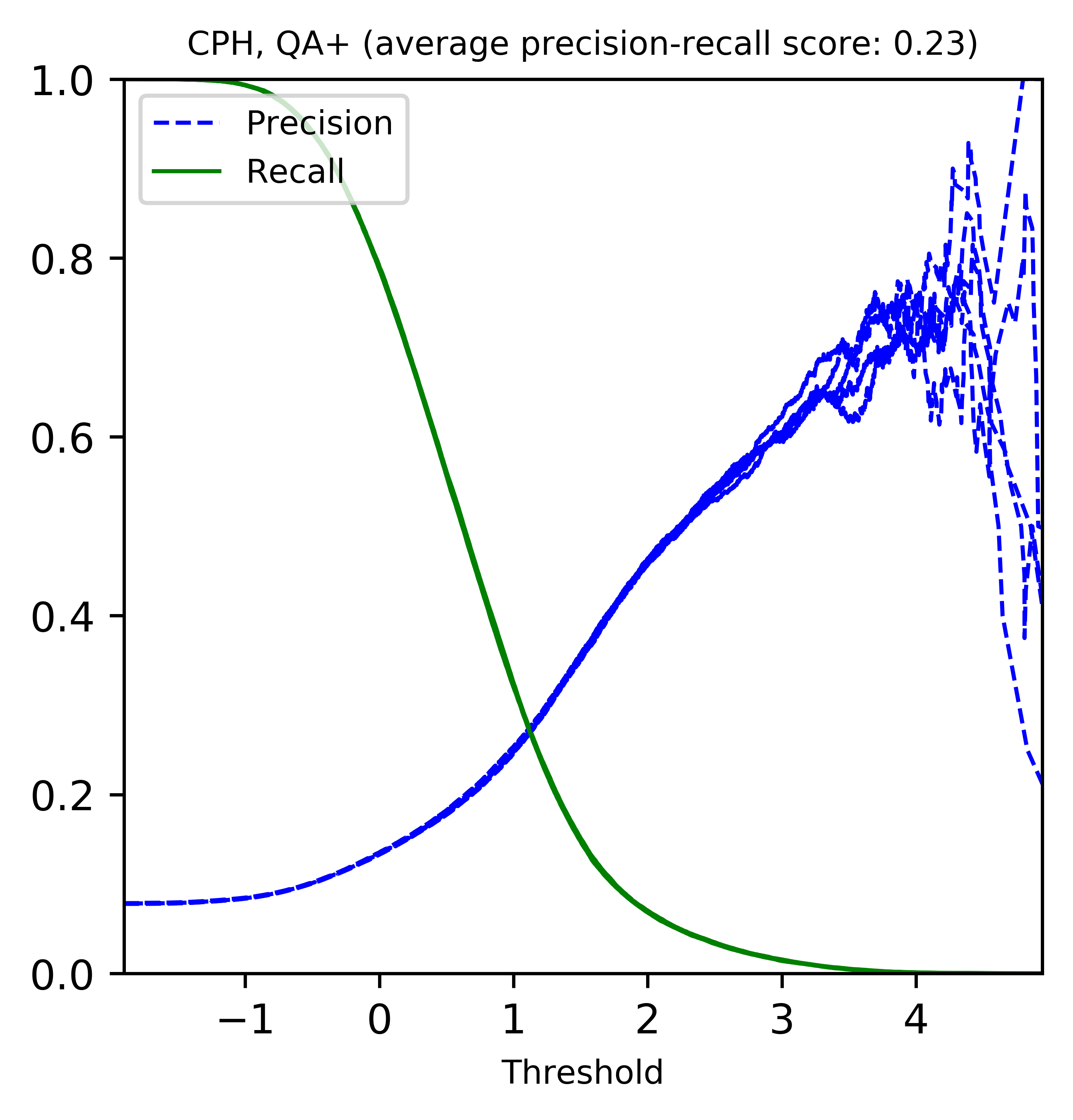 | 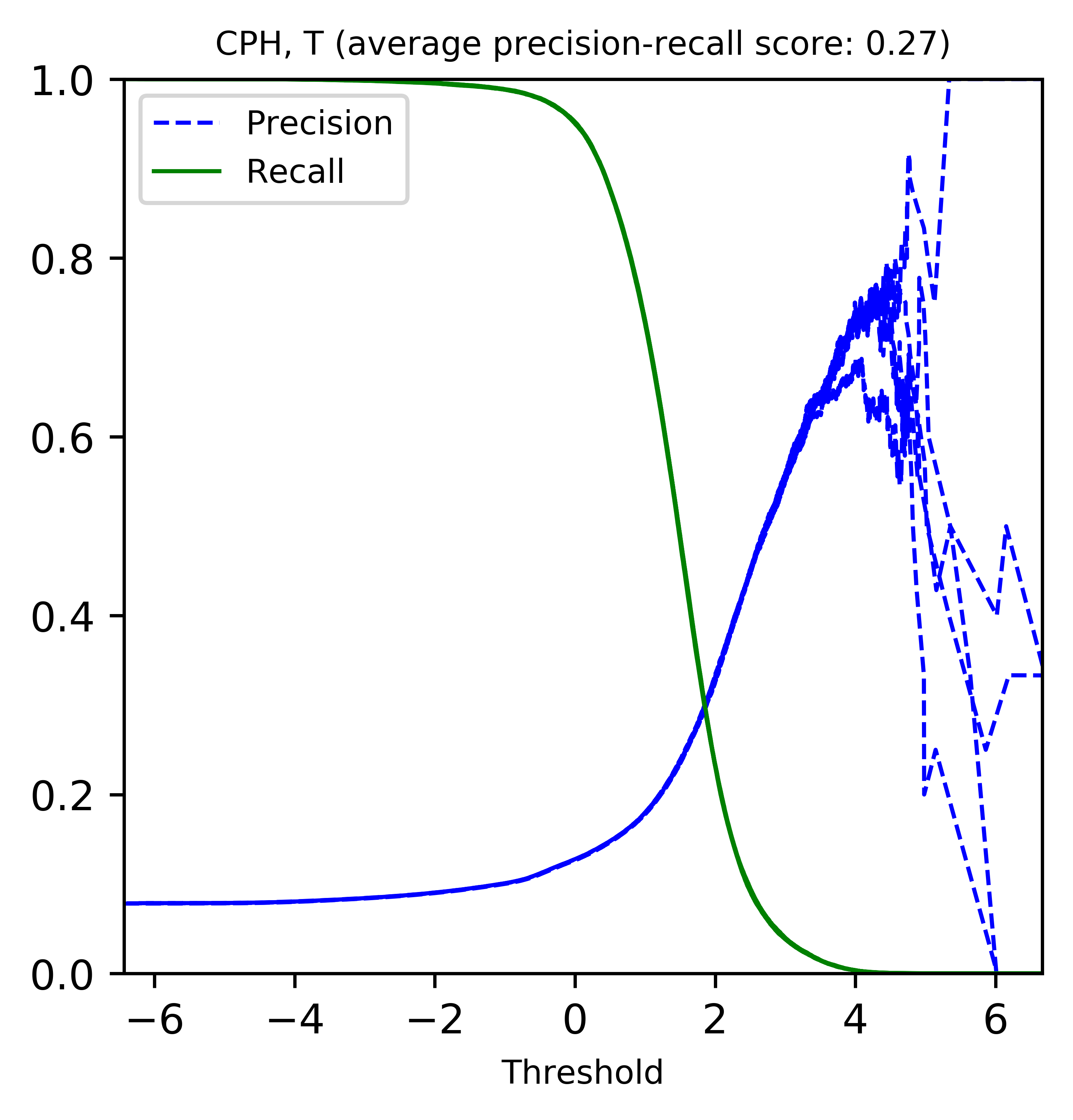 |
| RF | 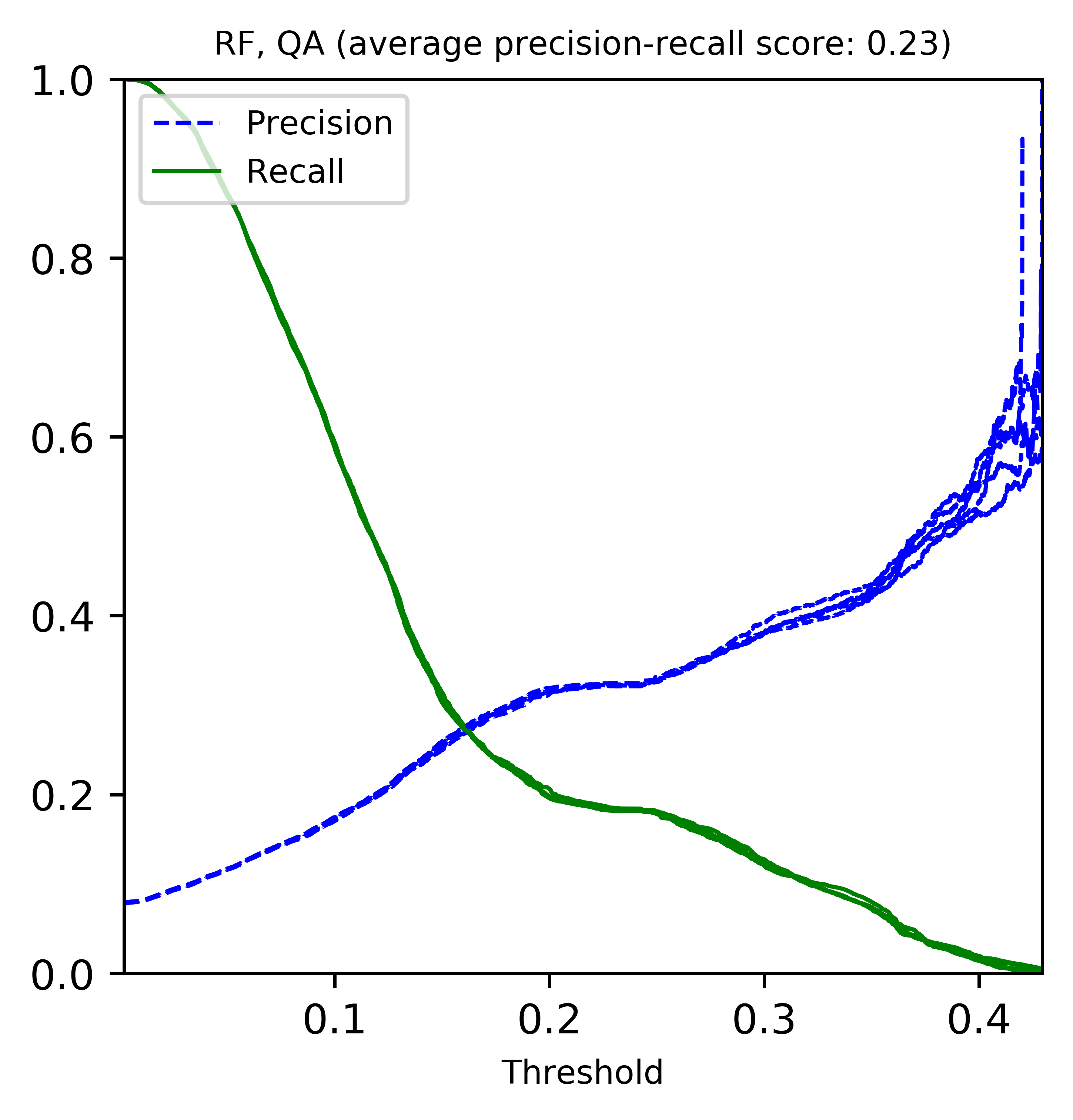 | 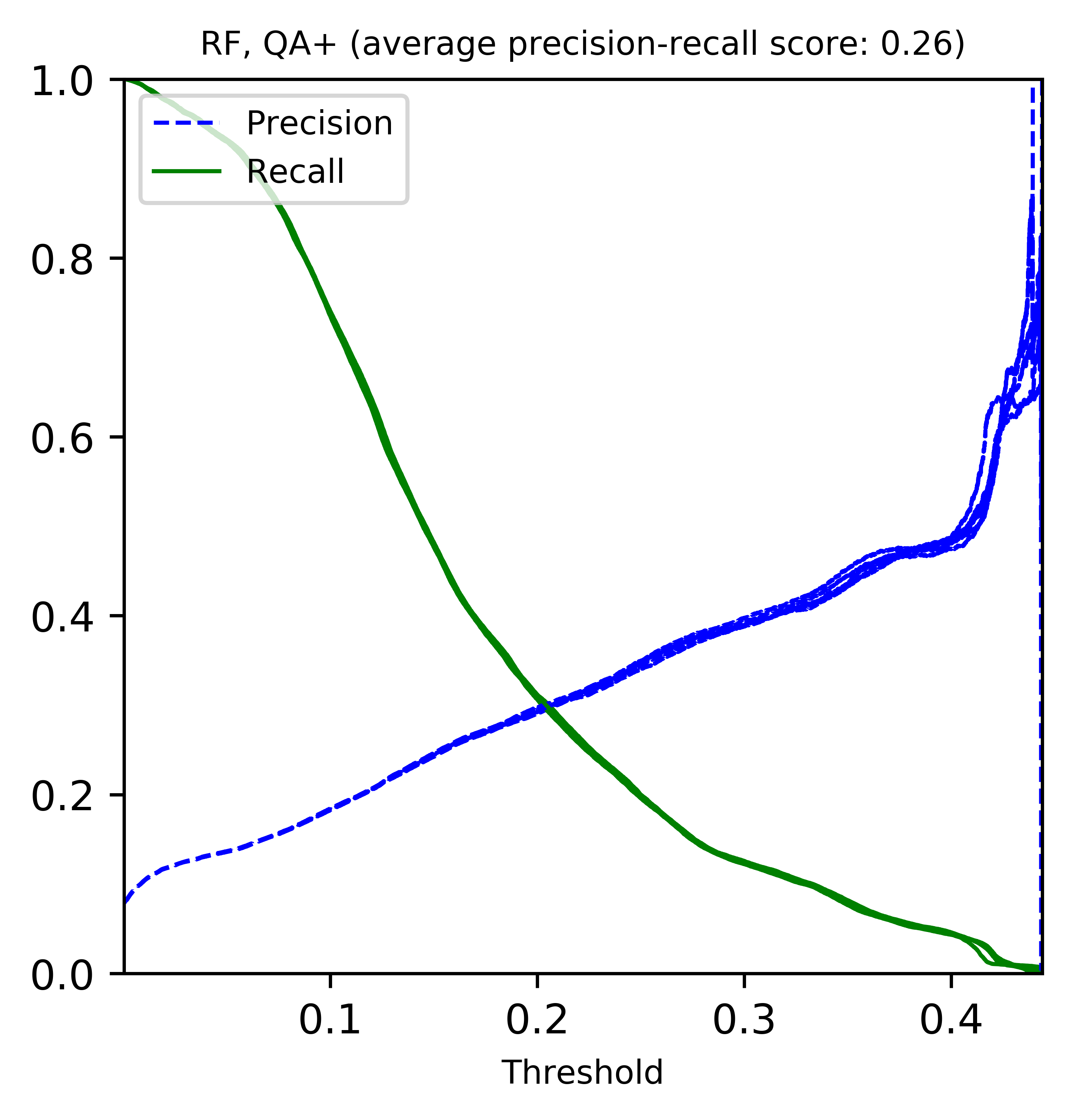 | 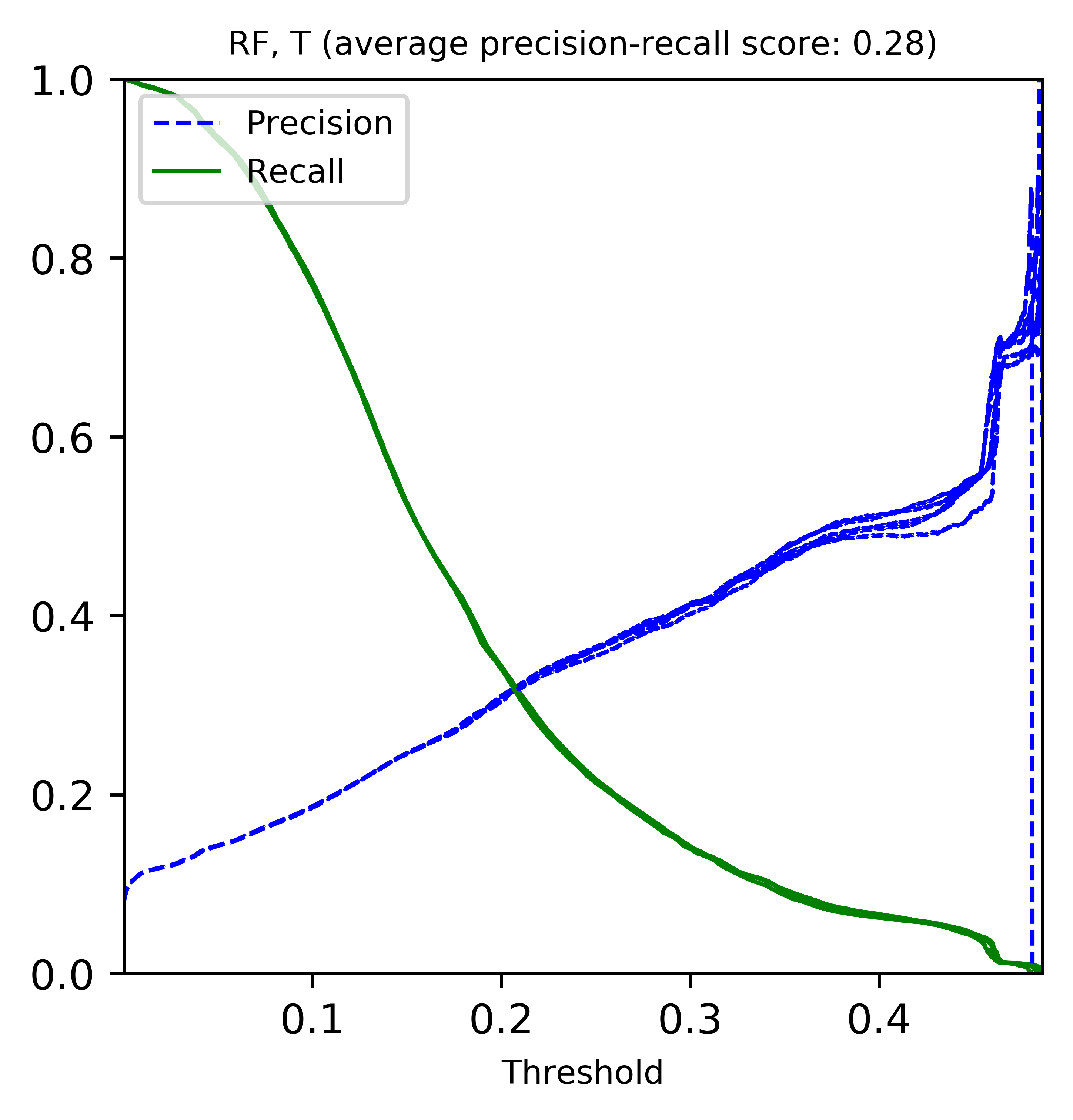 |
| GBC | 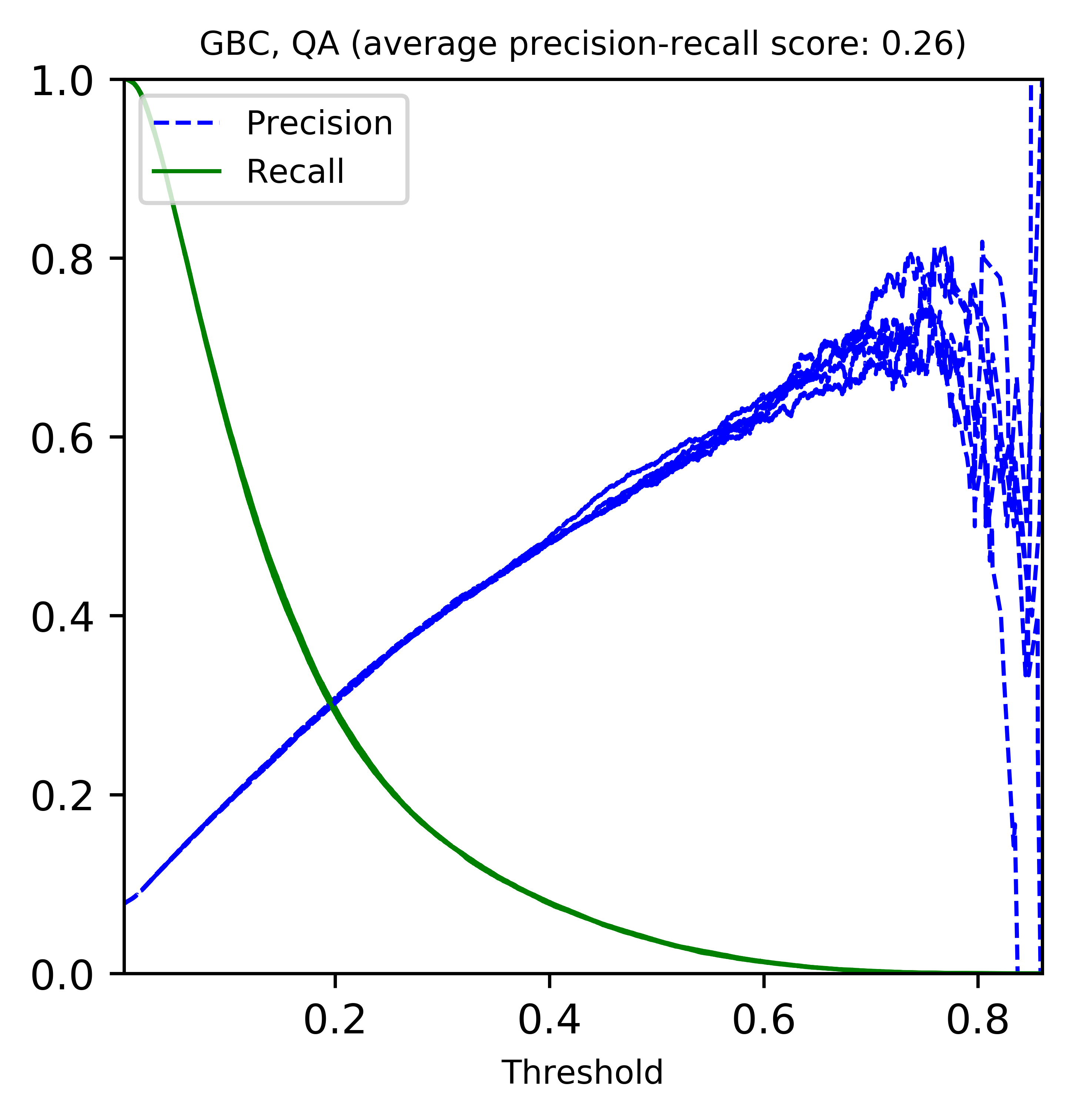 | 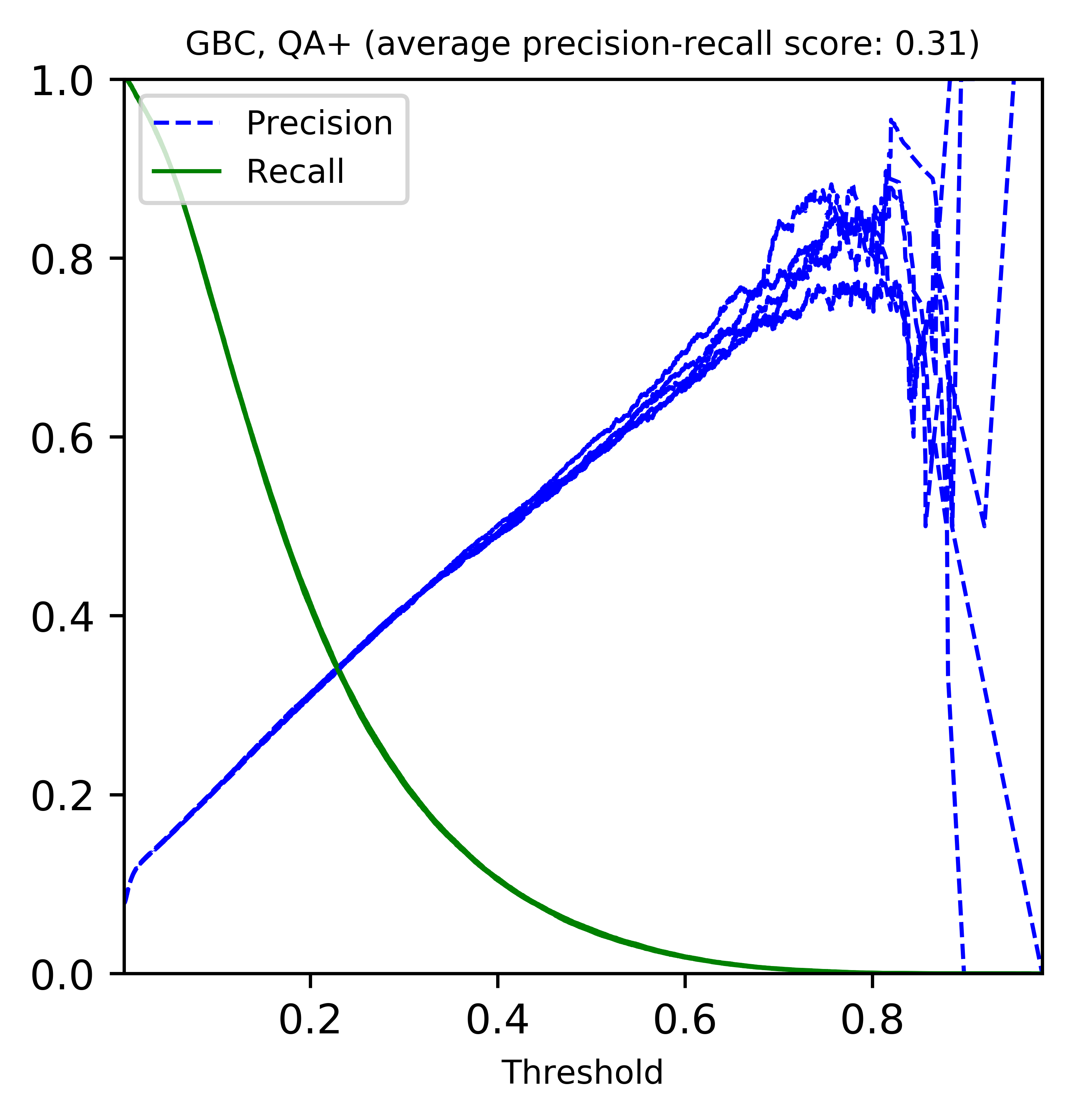 | 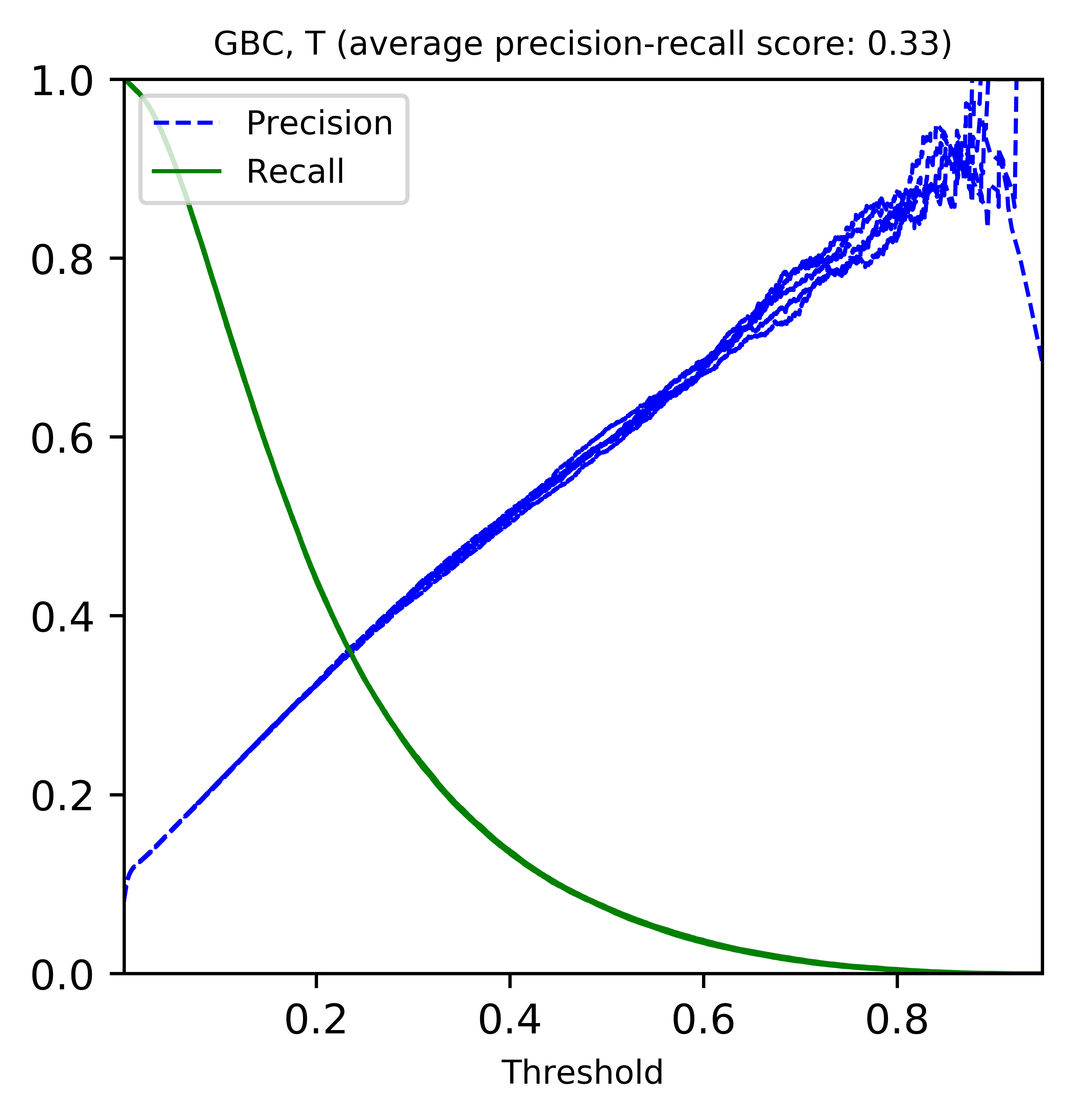 |

S1 Fig 4. Cross-validated precision and recall, by different predictor sets and modelling techniques (cohort: derivation). The values for all the folds are shown. These plots show how the tradeoff between precision and recall changes. Within any one model, you can also decide to emphasize either precision or recall.
