# Supplementary figures and images for: Predicting the risk of emergency admission with machine learning: Development and validation using linked electronic health records
Source: PLoS Med. 2018 Nov 20;15(11):e1002695. doi: 10.1371/journal.pmed.1002695 (PMC6245681; doi:10.1371/journal.pmed.1002695)

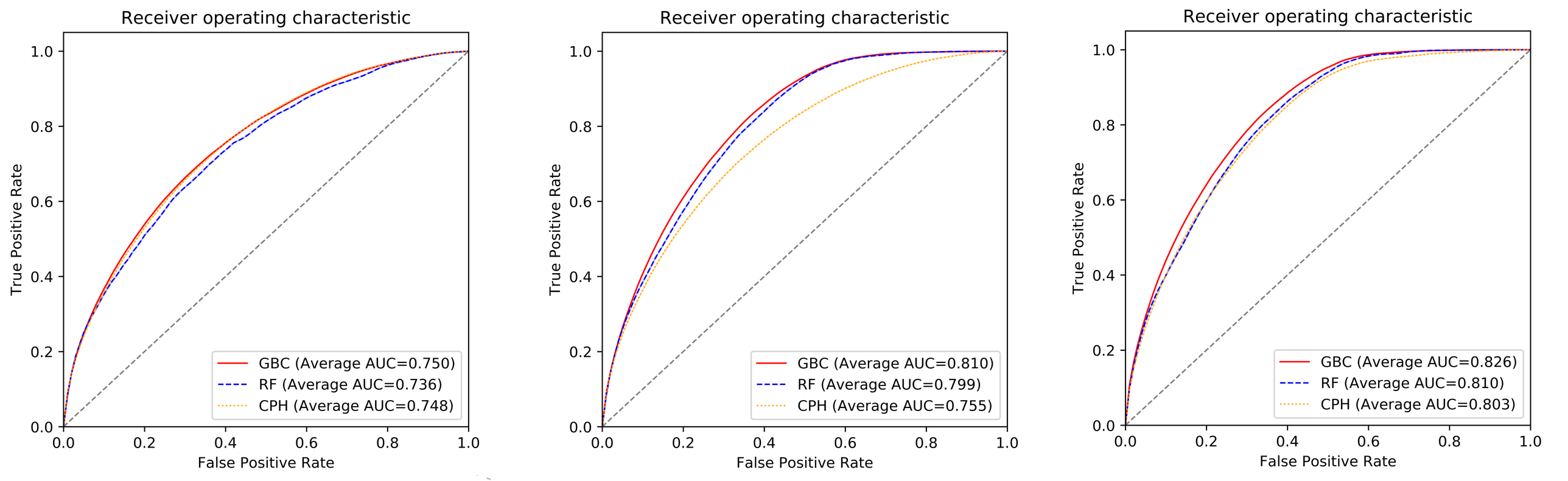

Supplement: S5 Fig — GBC constantly shows a better AUC regardless of the predictors used. (TIF) [file pmed.1002695.s005.tif]
