# Supplementary material for: Predicting the risk of emergency admission with machine learning: Development and validation using linked electronic health records
Source: PLoS Med. 2018 Nov 20;15(11):e1002695. doi: 10.1371/journal.pmed.1002695 (PMC6245681; doi:10.1371/journal.pmed.1002695)
Supplement: S1 Table — (DOCX) [file pmed.1002695.s010.docx]

| Predictors | Imputation model | GBC | RF | CPH |
| --- | --- | --- | --- | --- |
| QA | 1 | 0.768 | 0.745 | 0.733 |
|  | 2 | 0.777 | 0.755 | 0.742 |
|  | 3 | 0.777 | 0.752 | 0.740 |
| QA+ | 1 | 0.829 | 0.82 | 0.747 |
|  | 2 | 0.833 | 0.824 | 0.752 |
|  | 3 | 0.833 | 0.822 | 0.751 |
| T | 1 | 0.845 | 0.821 | 0.804 |
|  | 2 | 0.848 | 0.825 | 0.806 |
|  | 3 | 0.848 | 0.824 | 0.805 |
