# Supplementary material for: Predicting the risk of emergency admission with machine learning: Development and validation using linked electronic health records
Source: PLoS Med. 2018 Nov 20;15(11):e1002695. doi: 10.1371/journal.pmed.1002695 (PMC6245681; doi:10.1371/journal.pmed.1002695)
Supplement: S2 Table — (DOCX) [file pmed.1002695.s011.docx]

| Read Code | Med Code | Description |
| --- | --- | --- |
| 7M300 | 7242 | Emergency operation NOC |
| 8H1 | 11413 | Admit to intensive care unit |
| 8H1-1 | 8265 | Admit to I.T.U. |
| 8H11 | 38379 | Admit to cardiac ITU |
| 8H12 | 67786 | Admit to respiratory ITU |
| 8H13 | 65512 | Admit to neurological ITU |
| 8H14 | 66764 | Admit to metabolic ITU |
| 8H1Z | 22374 | Admit to intensive care unit NOS |
| 8H2 | 314 | Emergency hospital admission |
| 8H21 | 18512 | Admit medical emergency unspecified. |
| 8H22 | 11822 | Admit surgical emergency unspecified. |
| 8H23 | 7503 | Admit psychiatric emergency |
| 8H230 | 3488 | Emergency psychiatric admission MHA |
| 8H24 | 30002 | Admit geriatric emergency |
| 8H25 | 11963 | Admit paediatric emergency |
| 8H26 | 6109 | Admit gynaecological emergency |
| 8H27 | 24655 | Admit obstetric emergency |
| 8H28 | 23106 | Admit orthopaedic emergency |
| 8H29 | 11486 | Admit ENT emergency |
| 8H2A | 19209 | Admit trauma emergency |
| 8H2B | 25221 | Admit ophthalmological emergency |
| 8H2C | 29190 | Admit rheumatology emergency |
| 8H2D | 37543 | Admit dermatology emergency |
| 8H2E | 29836 | Admit neurology emergency |
| 8H2F | 12038 | Admit urology emergency |
| 8H2G | 46824 | Admit radiotherapy emergency |
| 8H2H | 35333 | Admit haematology emergency |
| 8H2I | 12243 | Admit plastic surgery emergency |
| 8H2J | 7059 | Admit diabetic emergency |
| 8H2K | 54704 | Admit oral surgical emergency |
| 8H2L | 29816 | Admit psychogeriatric emergency |
| 8H2M | 35328 | Admit renal medicine emergency |
| 8H2N | 28923 | Admit neurosurgical emergency |
| 8H2O | 29988 | Admit cardiothoracic emergency |
| 8H2P | 7058 | Emergency admission, asthma |
| 8H2Q | 30027 | Admit cardiology emergency |
| 8H2S | 32898 | Admit heart failure emergency |
| 8H2T | 95163 | Emergency voluntary psychiatric admission Mental Health Act |
| 8H2V | 95550 | Admit ischaemic heart disease emergency |
| 8H2W | 99761 | Admit vascular surgery emergency |
| 8H2Z | 6885 | Admit hospital emergency NOS |
| 8H63 | 13706 | Refer to casualty officer |
| 8H64 | 60874 | Refer to house officer |
| 8H65 | 25057 | Refer to hospital registrar |
| 8Hb | 48729 | Involuntary admission |
| 8HC | 1158 | Refer to hospital casualty |
| 8HC1 | 9409 | Refer to A. & E. department |
| 8HC2 | 6269 | Refer to hosp. eye casualty |
| 8HC3 | 32506 | Refer to hosp. paeds casualty |
| 8HCZ | 22296 | Refer to hospital casualty NOS |
| 8Hd1 | 102555 | Admission by accident and emergency doctor |
| 8Hd3 | 104164 | Admission by out of hours service doctor |
| 8Hd5 | 105263 | Admission to acute assessment unit |
| 8Hd6 | 105520 | Admission to stroke unit |
| 8HJA-1 | 1081 | Casualty self-referral |
| 8HJZ | 38567 | Self-referral to hospital NOS |
| 9b0K | 43828 | Hospital admission note |
| 9N04 | 8082 | Seen in emergency clinic |
| 9N19 | 140 | Seen in hospital casualty |
| 9Nk8 | 95995 | Seen in eye casualty department |
