# Supplementary material for: Predicting the risk of emergency admission with machine learning: Development and validation using linked electronic health records
Source: PLoS Med. 2018 Nov 20;15(11):e1002695. doi: 10.1371/journal.pmed.1002695 (PMC6245681; doi:10.1371/journal.pmed.1002695)
Supplement: S3 Table — (DOCX) [file pmed.1002695.s012.docx]

| **Parameter** | **Description** | **Selected Value** |
| --- | --- | --- |
| No. of trees | Number of estimators created | 800 |
| Max depth | Maximum depth of each tree | 5 |
| Min Sample Split | Minimum fraction of samples required to split a tree branch further | 0.01 |
| Max features | Maximum number of variables used in each tree | 0.5 |
