# Supplementary material for: Predicting the risk of emergency admission with machine learning: Development and validation using linked electronic health records
Source: PLoS Med. 2018 Nov 20;15(11):e1002695. doi: 10.1371/journal.pmed.1002695 (PMC6245681; doi:10.1371/journal.pmed.1002695)
Supplement: S4 Table — (DOCX) [file pmed.1002695.s013.docx]

|  |  | Derivation cohort | | Validation cohort | |
| --- | --- | --- | --- | --- | --- |
|  |  | Population | Incidence of outcome | Population | Incidence of outcome |
| Age | | | | | |
|  | 18-30 | 516,848 | 44,130 (8%) | 103,584 | 13,311 (12%) |
|  | 30-40 | 779,807 | 48,211 (6%) | 165,029 | 15,241 (9%) |
|  | 40-50 | 698,116 | 46,364 (6%) | 165,275 | 15,733 (9%) |
|  | 50-60 | 552,882 | 41,777 (7%) | 137,866 | 13,716 (9%) |
|  | 60-70 | 467,091 | 37,995 (8%) | 119,314 | 11,986 (10%) |
|  | 70-80 | 310,195 | 31,885 (10%) | 83,951 | 10,368 (12%) |
|  | 80-90 | 228,708 | 27,010 (11%) | 61,992 | 7,995 (12%) |
|  | 90-100 | 163,465 | 12,263 (7%) | 44,550 | 3,024 (6%) |
| Sex | | | | | |
|  | Female | 1,937,265 | 155,588 (8%) | 454,424 | 47,937 (10%) |
|  | Male | 1,812,667 | 137,543 (7%) | 432,941 | 44,179 (10%) |
| Strategic Health Authority | | | | | |
|  | South Central | 572,791 | 40,245 (7%) | - | - |
|  | South West | 558,036 | 54,689 (9%) | - | - |
|  | London | 817,870 | 62,732 (7%) | - | - |
|  | East of England | 540,346 | 29,239 (5%) | - | - |
|  | West Midlands | 518,586 | 39,908 (7%) | - | - |
|  | South East Coast | 591,472 | 59,028 (9%) | - | - |
|  | East Midlands | 150,831 | 7,290 (4%) | - | - |
|  | North West | - | - | 613,460 | 68,283 (11%) |
|  | North East | - | - | 89,004 | 11,060 (12%) |
|  | Yorkshire & The Humber | - | - | 184,901 | 12,773 (6%) |
| Overall | | | | | |
|  | - | 3,749,932 | 293,131 (7.82%) | 887,365 | 92,116 (10.38%) |
