# Supplementary material for: Predicting the risk of emergency admission with machine learning: Development and validation using linked electronic health records
Source: PLoS Med. 2018 Nov 20;15(11):e1002695. doi: 10.1371/journal.pmed.1002695 (PMC6245681; doi:10.1371/journal.pmed.1002695)
Supplement: S5 Table — (DOCX) [file pmed.1002695.s014.docx]

| Number of months after the baseline | Derivation cohort  n (%) | Validation cohort  n (%) |
| --- | --- | --- |
| 12 | 164,366 (4.38) | 54,953 (6.19) |
| 24 | 293,131 (7.82) | 92,116 (10.38) |
| 36 | 406,497 (10.84) | 120,133 (13.54) |
| 48 | 498,503 (13.29) | 141,781 (15.98) |
| 60 | 568,995 (15.17) | 156,465 (17.63) |
