# Supplementary material for: Predicting the risk of emergency admission with machine learning: Development and validation using linked electronic health records
Source: PLoS Med. 2018 Nov 20;15(11):e1002695. doi: 10.1371/journal.pmed.1002695 (PMC6245681; doi:10.1371/journal.pmed.1002695)
Supplement: S6 Table — (DOCX) [file pmed.1002695.s015.docx]

| rank | QA | QA+ | T |
| --- | --- | --- | --- |
| 1 | age | consultation_count^[[1]](#footnote-1)^ | consultation_duration^[[2]](#footnote-2)^ |
| 2 | cholesterol_ratio | age | age |
| 3 | haemoglobin | platelet_counts | consultation_count |
| 4 | SBP | haemoglobin_counts | SBP_since_last |
| 5 | last_year_admissions | gammagt_counts | admission_since_last |
| 6 | platelet | last_year_admissions | platelet_counts |
| 7 | bmi | aspartate_counts | aspartate_counts |
| 8 | IMD | bilirubin_counts | last_year_admissions |
| 9 | esr | haemoglobin | haemoglobin |
| 10 | region_South Central | IMD | gammagt_counts |
| 11 | statin | cholesterol_counts | pancreat_since_diag |
| 12 | region_London | bmi_counts | bilirubin_counts |
| 13 | smoking | esr_counts | IMD |
| 14 | hypertension | region_London | consultation_since_last |
| 15 | ethnicity_Unknown | region_South West | falls_since_diag |
| 16 | region_West Midlands | region_South Central | region_London |
| 17 | region_South West | bmi | region_South Central |
| 18 | asthma_COPD | SBP | vte_since_diag |
| 19 | anticoag | cholesterol_ratio | SBP |
| 20 | alcohol | region_West Midlands | region_South East Coast |

1. ‘count’ refers to the number of times a variable was recorded. [↑](#footnote-ref-1)
2. Total time spent in GP. [↑](#footnote-ref-2)
