# Supplementary material for: Predicting the risk of emergency admission with machine learning: Development and validation using linked electronic health records
Source: PLoS Med. 2018 Nov 20;15(11):e1002695. doi: 10.1371/journal.pmed.1002695 (PMC6245681; doi:10.1371/journal.pmed.1002695)
Supplement: S7 Table — (DOCX) [file pmed.1002695.s016.docx]

| rank | QA | QA+ | T |
| --- | --- | --- | --- |
| 1 | last_year_admissions | consultation_count | consultation_count |
| 2 | liver_function_test | last_year_admissions | consultation_duration |
| 3 | ethnicity_Unknown | haemoglobin_counts | last_year_admissions |
| 4 | platelet | platelet_counts | admission_since_last |
| 5 | nsaids | bilirubin_counts | SBP_last |
| 6 | age | ethnicity_Unknown | consultation_since_last |
| 7 | antidepressant | age | ethnicity_Unknown |
| 8 | statin | IMD | haemoglobin_since_last |
| 9 | haemoglobin | haemoglobin | platelet_counts |
| 10 | ethnicity_White | ethnicity_White | IMD |
| 11 | cholesterol_ratio | platelet | age |
| 12 | region_South East Coast | nsaids | platelet_since_last |
| 13 | IMD | region_South East Coast | falls_since_diag |
| 14 | corticosteroids | bmi_counts | bmi_since_last |
| 15 | anticoag | liver_function_test | SBP |
| 16 | region_East of England | cholesterol_counts | ethnicity_White |
| 17 | SBP | region_East of England | nsaids |
| 18 | region_East Midlands | gammagt_counts | bilirubin_counts |
| 19 | region_South West | gender | region_South East Coast |
| 20 | gender | anticoag | cvd_since_diag |
